# Overexpression of SERPINA3 inhibits castration-resistant prostate cancer progression by enhancing M1 macrophage recruitment via CXCL2 upregulation

**DOI:** 10.1590/1414-431X2025e14445

**Published:** 2025-05-09

**Authors:** Jianbing Xie, Qiren Chen, Lixian Li, Jinyu Liu

**Affiliations:** 1 Department of Urology, Affiliated Hospital of Putian University, Putian, Fujian, China; 2 Department of Breast Surgery, Affiliated Hospital of Putian University, Putian, Fujian, China

**Keywords:** CRPC, SERPINA3, CXCL2, Macrophage, Tumor microenvironment

## Abstract

The primary objective of the present study was to identify differentially expressed genes (DEGs) associated with castration-resistant prostate cancer (CRPC) to verify the potential mechanism of CRPC progression. DEGs from CRPC datasets were filtered with a P<0.05 and Spearman correlation coefficient ≥0.3. Serpin peptidase inhibitor, clade A member 3 (SERPINA3), was uniquely present in three CRPC datasets, and its low expression in CRPC was confirmed in cell lines and tissues. Colony formation, transwell assays, and subcutaneous tumor formation experiments in mice demonstrated that overexpression of SERPINA3 may significantly inhibit the proliferation and invasion of PC3 cells. Mechanistic studies revealed that, in prostate cancer (PCa), SERPINA3 can activate the interleukin (IL)-17 and tumor necrosis factor (TNF)α signaling pathways by promoting the expression of CXC chemokine ligand 2 (CXCL2), thereby increasing the recruitment of M1 macrophages into the tumor microenvironment and inhibiting the progression of PCa. The current results indicated that the expression of SERPINA3 may be negatively correlated with CRPC, and it could promote the M1 polarization of macrophages and inhibit the progression of CRPC by increasing the expression of CXCL2.

## Introduction

Prostate cancer (PCa) is a common malignant tumor in men, the incidence rate and mortality rate of which increases every year (1). Notably, ∼70% of patients with PCa in China have advanced stage cancer at the time of initial diagnosis, which poses a challenge for treatment (2). Although treatment of early stage cancer with castration can benefit some patients with invasion and metastasis, a large number of patients with PCa will eventually form castration-resistant PCa (CRPC), which often leads to death (3). Therefore, it is essential to investigate the mechanisms underlying the occurrence and development of CRPC.

Serpin peptidase inhibitor, clade A member 3 (SERPINA3), also known as α-1-antichymotrypsin, is a member of the serine protease inhibitor family, which is encoded by the SERPINA3 gene located in the 14q32.13 region of chromosome 14. The primary function of SERPINA3 is to inhibit serine proteases, such as plasmin, tissue proteinase G, and mast cell protease, by binding them in a stable complex, thereby preventing their proteolytic activity and influencing the composition of the extracellular matrix (4,5). SERPINA3 plays a critical role in tumorigenesis and progression. In previous studies, SERPINA3 has been identified as an important diagnostic marker for various types of cancer, including liver cancer, breast cancer, and colorectal cancer (6-8). However, the functional role of SERPINA3 in PCa has not been extensively investigated. In bone metastases of PCa, overexpression of SERPINA3 has been detected via hematoxylin and eosin staining, leading to enhanced osteogenic differentiation. It has been hypothesized that the overexpression of SERPINA3 may inhibit bone destruction (9). Notably, the impact of SERPINA3 expression on the progression of PCa remains to be fully elucidated.

In this study, we investigated whether the expression of SERPINA3 in prostate cancer cells is associated with the progression of CRPC. We established a stable cell line overexpressing SERPINA3 in PC3 cells and validated the function of SERPINA3 through cell cloning, invasion, migration assays, and subcutaneous tumor experiments. Using bioinformatics techniques, we conducted in-depth analysis and validation of the relationship between SERPINA3 and CXCL2 in the tumor microenvironment, elucidating their role in macrophages. This provided new insight into the molecular mechanisms underlying CRPC progression.

## Material and Methods

### Bioinformatics analysis

The transcriptome sequencing data from the GSE32269, GSE101607, and GSE109708 datasets were filtered using a threshold of P<0.05 and Spearman coefficient ≥0.3 to obtain differentially expressed genes (DEGs), followed by Venn diagram intersection analysis. The correlation between SERPINA3 expression and the progression of PCa was investigated using various databases in the Prostate Cancer Database (https://bioinformatics.cruk.cam.ac.uk/apps/camcAPP/), including Michigan 2012, Cambridge, and Memorial Sloan Kettering Cancer Center (MSKCC). The Cancer Genome Atlas (TCGA), MSKCC, and Cambridge databases were used to analyze the association of SERPINA3 expression with patient survival in PCa. The University of Alabama at Birmingham Cancer (UALCAN) data analysis portal (https://ualcan.path.uab.edu/index.html), MSKCC, and Cambridge databases were used for detecting the relationship between the expression of SERPINA3 and the Gleason score of patients with PCa. The Human Protein Atlas (HPA) database (https://www.proteinatlas.org/) was utilized to evaluate the staining patterns of SERPINA3 in tumor tissues of patients with different grades of PCa. The expression of SERPINA3 and its association with the TNM staging of patients with PCa was validated using the LinkedOmics and UALCAN databases. Timer2.0 (http://timer.cistrome.org/) and cBioPortal (https://www.cbioportal.org/) databases were employed to analyze the relationship between the expression of SERPINA3 and M1/M2 macrophage infiltration/markers in a PCa cohort. Kyoto Encyclopedia of Genes and Genomes (KEGG) and Venn enrichment were performed using the OECloud tools (https://cloud.oebiotech.com).

### Cell culture and construction of CRPC cell lines

The human PCa cell lines LNCAP, VCAP, C4-2B, PC3, and DU145 and the human monocytic leukemia cell line THP-1 were purchased from American Type Culture Collection. LNCAP, VCAP, and C4-2B cell lines were cultured in high-glucose DMEM (Gibco, USA) supplemented with 10% fetal bovine serum (Gibco) and 1% penicillin/streptomycin. THP-1, PC3, and DU145 cell lines were cultured in RPMI (Roswell Park Memorial Institute)-1640 medium (Gibco) supplemented with 10% fetal bovine serum (Gibco). The cells were maintained at 37°C in a humidified atmosphere containing 5% CO_2_. All cell lines were authenticated through short tandem repeat analysis. The androgen-dependent PCa cell line LNCAP was cultured in charcoal-treated fetal bovine serum (Cat. No. 0702-TXF; Wenrenbio, China), and cell proteins were then extracted at different time points and cryopreserved.

### Establishment of stable cell lines overexpressing SERPINA3

PC3 cells were seeded onto a 24-well plate until they reached ∼70% confluence. pLenti-CMV-Puro-GFP-SERPINA3 plasmid was derived from pLenti-CMV-Puro-GFP vector by inserting SERPINA3. The pLenti-CMV-Puro-GFP vector was used as the negative control. Subsequently, 10 μL Vector or SERPINA3-overexpressing lentiviral particles (Beijing Tsingke Biotech Co., Ltd., China) were added and mixed well. After 24 h, the medium was replaced and supplemented with puromycin for selection, until stable cells exhibiting GFP fluorescence labels were observed. Subsequently, western blotting and quantitative qPCR experiments were performed to validate the efficiency of SERPINA3 overexpression.

### Colony formation assay

PC3 cells from the Vector group and the stable SERPINA3-overexpressing group were digested with trypsin and resuspended in serum-containing medium. After centrifugation at 200 *g* at room temperature for 3 min, the cell pellet was resuspended in 1 mL medium. The cell count was determined, and 100-200 cells were then seeded onto each well of a 6-well plate, with 3 wells for both the Vector and SERPINA3-overexpressing cell groups. After the single cells formed colonies, and the number of cells in each colony was observed to be >50 under a microscope, they were fixed with 4% paraformaldehyde for 20 min. Finally, the colonies were stained with crystal violet for 5 min, excess crystal violet was washed off, the colonies were air-dried, and images were captured.

### Cell counting kit 8 (CCK8) assay

PC3 cells from the Vector group and the stable SERPINA3-overexpressing group were seeded onto 96-well plates (2,000 cells). At 24, 48, and 72 h after seeding, CCK8 (Cat. No. G4103; Servicebio, China) solution was added at a ratio of 1:9 into the 96-well plates, and the cells were incubated for 2 h in a cell culture incubator. Subsequently, absorbance values at a wavelength of 450 nm were measured using a spectrometer.

### Transwell assay

A total of 2,000-3,000 PC3 cells from the Vector group and the stable SERPINA3-overexpressing group were harvested and transferred to the upper transwell chamber (pore size, 8 μm). Then, RPMI-1640 medium was added to the upper chamber to increase the volume to 200 μL, and 600 μL complete medium was added to the lower chamber for 18 h. After washing the transwell chambers twice with PBS, cells were fixed with 4% paraformaldehyde for 20 min and then stained with crystal violet for 8 min. Subsequently, the cells were observed and images were captured under a microscope (Olympus, BX53, Olympus, Japan).

### ELISA

Supernatants were collected from cells in the Vector and stable SERPINA3-overexpressing groups and were concentrated using a freeze-dryer. Equal volumes of chromogenic agent A and chromogenic agent B were then mixed, protected from light, and shaken well. Subsequently, 100 μL of this mixture, prepared fresh each time, was added to each well. A dilution buffer was prepared by mixing deionized water to achieve a 1X working concentration.

The detection antibody powder was centrifuged (at 12,000 *g* and 4°C for 5 min), then resuspend in a working concentration of dilution buffer. The lyophilized standard was reconstituted according to the instructions provided for preparing standard curve dilutions using dilution buffer. Subsequently, ELISA was performed as follows: prepared samples (100 μL) were added to each well of a 96-well plate, which was incubated at 37°C in a cell incubator for 1 h. The plate was washed with washing solution and 100 μL freshly prepared enzyme-labeled antibody was added to each well. The plate was incubated for 0.5 h and then washed three times, after which, the substrate was developed by adding 100 μL TMB (3,3',5,5'-tetramethylbenzidine) solution to each well for 0.5 h. Finally, the reaction was terminated with a stop solution and absorbance was measured at 450 nm using a microplate reader. The ELISA kits used were provided by MLbio company (China), with the following Cat. No.: ML058180 (CXCL2), ML057794 (CXCL1), ML060216 (CXCL6), ML057400 (CCL2), and ML060006 (CCL2O). The CXCL3 kit was provided by Ybio company (China), with the Cat. No. KKX-16405R.

### Xenograft mouse model

For the Vector and SERPINA3-overexpressing groups, ten male nude mice (age 5 weeks) were randomly divided into negative control and experimental groups. After 1 week of adaptation to the SPF environment, a subcutaneous injection of cells was prepared for each mouse using 200 μL cell suspension containing ∼1×10^7^ cells. The injection site of the tumor cells was the right axillary region of the mouse. Starting from day 3, the longest and shortest diameters of the tumors were recorded every 3 days until day 30, and the tumor volume (V) was calculated using the formula V = (W^2^ × L) / 2, where L is the longest diameter of the tumor and W is the shortest diameter. The mouse tumors were measured to ensure that the maximum diameter did not exceed 1.5 cm. At the end of the experiment, mice were euthanized by cervical dislocation to ensure immediate loss of consciousness and cessation of breathing, and the subcutaneous tumors were excised and weighed. The tumor tissues were then embedded into wax blocks for subsequent experiments.

### Immunohistochemistry and immunofluorescence

Patients who underwent castration therapy at Putian University Affiliated Hospital between January 2020 and December 2023 and whose postoperative pathological diagnosis confirmed castration-resistant prostate cancer were selected. Tumor tissue samples from patients were collected post-surgery, fixed in formalin, and embedded in paraffin blocks by the pathology department. The human prostate cancer tissues and mouse subcutaneous tumor tissues embedded in paraffin were sectioned into 5-μm-thick slices. Microwave repair was performed for 3 min, followed by washing with PBS and fetal bovine serum (Gibco) blocking for 1 h. The corresponding primary antibody was added under dark conditions and incubated overnight at 4°C. After washing with PBS, the next day, the secondary antibody was added and incubated for 30 min at room temperature. DAB staining was used for immunohistochemistry to observe staining. Subsequently, the slides were placed in a solution of hematoxylin for 1 min, washed with running water, and air-dried before being covered with a cover slip. Immunofluorescence steps were performed in the same way as immunohistochemistry, with DAPI added for nuclear staining. Finally, the slides were sealed with a mounting medium containing an anti-fluorescence quencher (Cat. No. G1401; Servicebio, China).

### Macrophage polarization and transwell assay

THP-1 cells were seeded onto a 6-well plate and induced with 100 ng/mL phorbol myristate acetate (PMA; Cat. No. HY-18739; MCE) to generate M0 macrophages, followed by induction with 20 ng/mL lipopolysaccharide (LPS; Cat. No. HY-D1056; MCE) for 12 h to generate M1 macrophages. Subsequently, cell counting was performed, and 50,000 cells were placed in the upper chamber and diluted to 200 μL with RPMI-1640. The lower chamber was filled with conditioned medium containing wild-type (WT), Vector, and SERPINA3-overexpressing PC3 cells. The chambers were incubated for 24 h and then processed according to the aforementioned transwell assay steps.

### Flow cytometry

After washing the adherent cells with PBS and thoroughly resuspending them, cells were washed three times with PBS and then incubated with the appropriate proportions of antibodies for flow cytometry. The antibodies used were as follows: anti-human CD68 antibody (Cat. No. 11-0689-41; Thermo Fisher, USA) and anti-human CD86 antibody (Cat. No. 12-0869-41; Thermo Fisher). Finally, flow cytometry analysis was performed using a flow cytometer (BD-LSRFortessa™, USA).

### Reverse transcription-qPCR

After removing the cell culture medium, cells were washed three times with PBS and total RNA was extracted using TRIzol reagent (Cat. No. 317908; Ambion, USA). The RNA was then reverse transcribed into cDNA using PrimeScript reverse transcriptase (Cat. No. DRR047A; TaKaRa, China). For qPCR, SYBR Green Master Mix (Cat. No. A25742; Thermo Fisher Scientific, China), and primers synthesized by Sangon Biotech were used on a Bio-Rad CFX Connect real-time system (USA). mRNA expression levels were detected using the 2^-ΔΔCq^ method and the expression level of glyceraldehyde-3-phosphate dehydrogenase (GAPDH) was used for normalization. The relevant primer sequences are shown in Table 1.

**Table 1:** Primer sequences used in this research.

	Forward primers	Reverse primers
SERPINA3	GTGTGGAGTGTGAGGGAGTTG	TCGTCAAGTGGGCTGTTAGG
CXCL2	AAAGCTTGTCTCAACCCCG	GGTCAGTTGGATTTGCCATTTT
CXCL1	CCCAAACCGAAGTCATAGCCA	CCCAAACCGAAGTCATAGCCA
CXCL3	CGAAAAGATACTGAACAAGGGGAGC	CATTTTCAGCTCTGGTAAGGGC
CXCL6	AGCAAGTTTGTCTGGACCCG	CAGTTTTTCTTGTTTCCACTGTCCA
CCL2	GTCCCAAAGAAGCTGTGATCTTCAA	TGGGTTGTGGAGTGAGTGTT
CCL20	TATTGTGCGTCTCCTCAGTAAA	AAGTGAAACCTCCAACCCCA
GAPDH	GGATTTGGTCGTATTGGG	GGAAGATGGTGATGGGATT

### Western blotting

After washing the cells three times with PBS, cell proteins were extracted using RIPA lysis buffer (Cat. No. P0013; Beyotime, China) and quantified using the BCA assay kit (Cat. No. P0010; Beyotime), according to the manufacturer's instructions. The proteins were loaded onto gels and separated by SDS-PAGE, before being transferred onto a PVDF membrane (Cat. No. 1620177; Bio-Rad, USA) using a semi-dry transfer apparatus (Bio-Rad). The membrane was blocked at room temperature for 10 min with a protein-free rapid sealing solution (Cat. No. G2052; Servicebio), followed by overnight incubation at 4°C with primary antibodies. The membrane was washed three times with PBS-Tween (PBST) for 15 min/wash and then incubated with the corresponding secondary antibodies (1:5,000; Cat. No. 511203 and 1:5,000; Cat. No. 550010; Zenbio, China) for 1 h at room temperature. The membrane was washed a further three times with PBST for 15 min/wash, and protein signals were detected using super-sensitive ECL Western HRP substrate (Cat. No. 17047; Zenbio) and a Bio-Rad ChemiDoc MP system (170-8280). The antibodies used in this experiment include: SERPINA3 (1:1,000; Cat. No. ab205198; Abcam, USA), androgen receptor (AR; 1:1,000; Cat. No. R380686; Zenbio), and GAPDH (1:5,000; Cat. No. ab8245; Abcam).

### Statistical analysis

All data presented in this study are representative of at least three independent replicates. Quantitative data are reported as means±SE and were analyzed using GraphPad Prism 8 software. Differences between the two groups were compared using unpaired Student's *t*-test. P<0.05 was considered to indicate a statistically significant difference.

## Results

### SERPINA3 expression was negatively associated with CRPC

**Figure 1:**
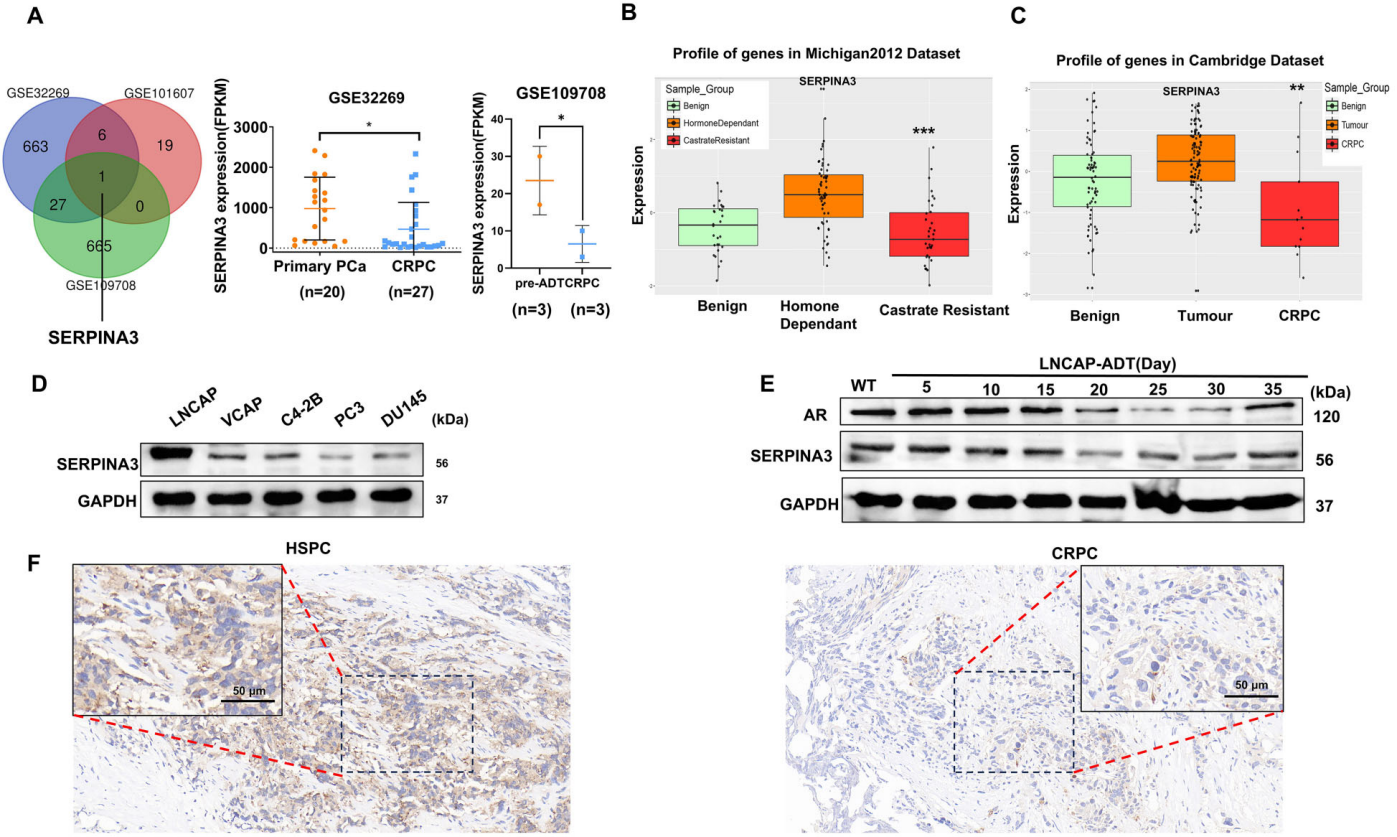
Negative association between SERPINA3 expression and castration-resistant prostate cancer (CRPC). **A**, Differentially expressed genes identified by Venn analysis in the three CRPC-related datasets (GSE101607, GSE32269, and GSE109708) and the expression of SERPINA3 in GSE32269 and GSE109708 datasets. **B**, Profile of SERPINA3 expression in the Michigan 2012 PCa dataset. **C**, Profile of SERPINA3 expression in Cambridge 2012 PCa dataset. **D**, Expression of SERPINA3 in five PCa cell lines (LNCAP, VCAP, C4-2B, PC3, and DU145) detected by western blotting. **E**, Protein expression of SERPINA3 was detected in LNCAP cells treated with androgen-deprived medium for different durations. **F**, Representative images of SERPINA3 staining in tissue samples from patients with hormone therapy-sensitive PCa as well as patients with CRPC (n=3:3). Data are reported as means±SE and were analyzed by two-tailed unpaired Student's *t*-test. *P<0.05, **P<0.01, ***P<0.001. Scale bar, 50 μm. AR: androgen receptor.

Three CRPC datasets were analyzed in the GEO database, with P<0.05 and fold change>|1| set as the screening criteria. DEGs of the three datasets were subjected to Venn intersection, and only the DEG SERPINA3 was found to exist in all three datasets. Notably, the CRPC group exhibited low expression of SERPINA3 in the GSE32269 and GSE109708 datasets (Figure 1A). Verifying the expression of SERPINA3 in hormone-sensitive and CRPC samples in the human PCa datasets, it was revealed that SERPINA3 expression was significantly downregulated in CRPC samples in Michigan 2012 and Cambridge datasets (Figure 1B and C). Similarly, the expression of SERPINA3 in hormone-sensitive PCa cell lines LNCAP was significantly upregulated compared with that in CRPC cell lines (VCAP, C4-2B, PC3, and DU145) (Figure 1D). During the process of androgen deprivation in LNCAP cells cultured *in vitro*, the expression of SERPINA3 gradually decreased, while the expression of AR gradually decreased in the early stage of castration treatment and significantly increased on the 35th day (Figure 1E). Furthermore, downregulation of SERPINA3 expression in CRPC was detected compared within hormone-sensitive PCa (Figure 1F). These results indicated that downregulation of SERPINA3 expression was negatively associated with CRPC.

### Expression of SERPINA3 was significantly negatively correlated with the clinical Gleason score of Pca

**Figure 2:**
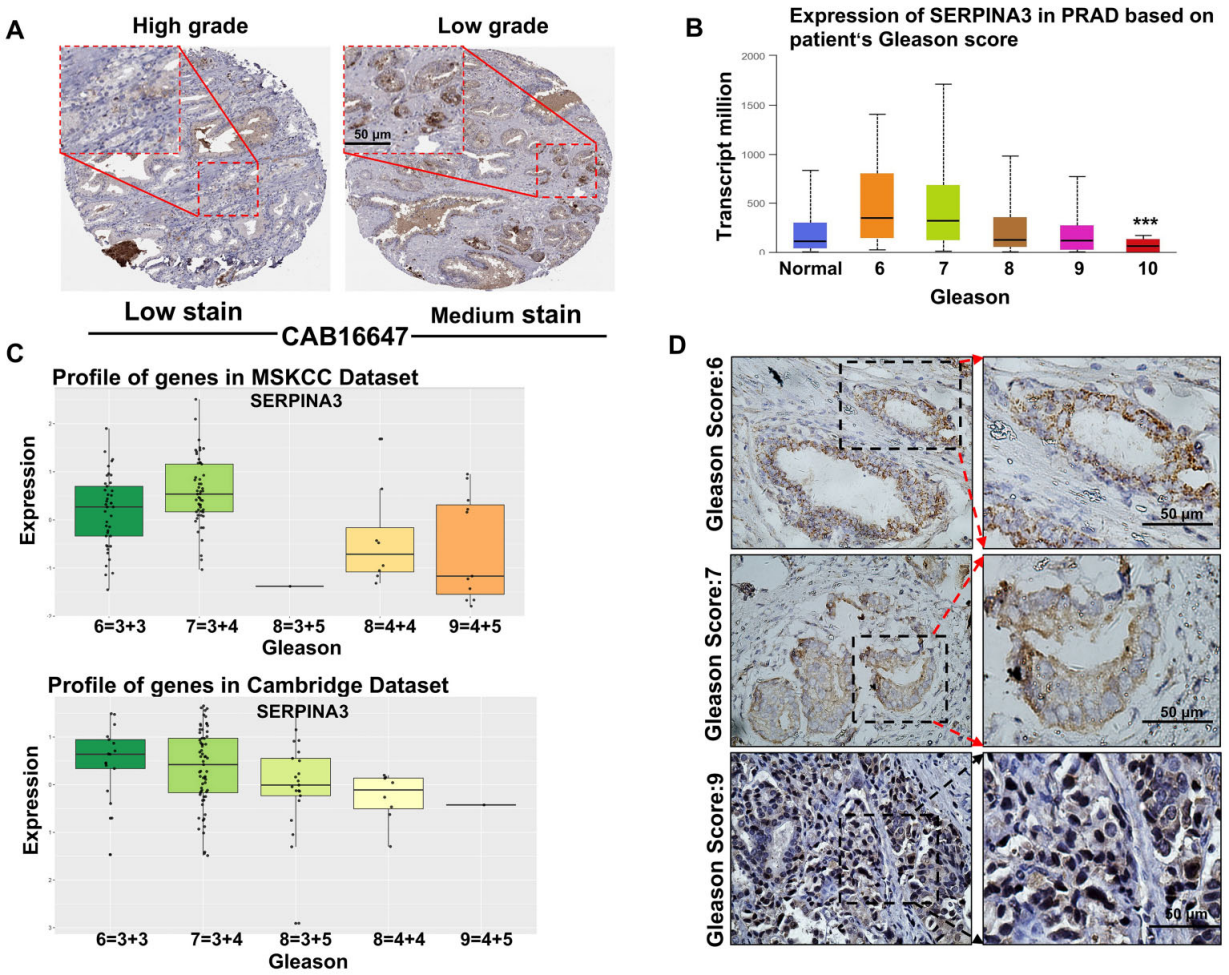
Expression of SERPINA3 is negatively correlated with the progression of prostate cancer. **A**, Staining pattern of SERPINA3 in low-grade and prostate cancer pathological tissues in the Human Protein Atlas (HPA) database. **B**, Expression of SERPINA3 in the TCGA-prostate adenocarcinoma (PRAD) database based on patient Gleason score. **C**, Expression of SERPINA3 in MSKCC and Cambridge datasets based on patient Gleason score. **D**, Expression of SERPINA3 in tumor tissues of patients with prostate cancer and different Gleason scores. Data are reported as median and interquartile range. ***P<0.001; one-way ANOVA. Scale bar, 50 μm.

To investigate whether SERPINA3 is associated with the progression of PCa, the HPA database was used; the results showed that the expression of SERPINA3 in low-grade PCa tissue was higher than that in high-grade PCa tissue using the antibody CAB16647 (Figure 2A). In addition, the UALCAN, which covered TCGA data of patients with PCa, verified the negative relationship between SERPINA3 expression and Gleason score (Figure 2B). Similarly, the expression of SERPINA3 was confirmed to be negatively correlated with Gleason score in both the MSKCC and Cambridge PCa datasets (Figure 2C). It was also confirmed in collected clinical tissue samples of patients with PCa and varying Gleason scores that the expression of SERPINA3 decreased with increasing Gleason score (Figure 2D). These results further confirmed the correlation between SERPINA3 and PCa malignancy from the perspective of clinical data.

### Low SERPINA3A expression had a significant impact on PCa metastasis and patient survival

**Figure 3:**
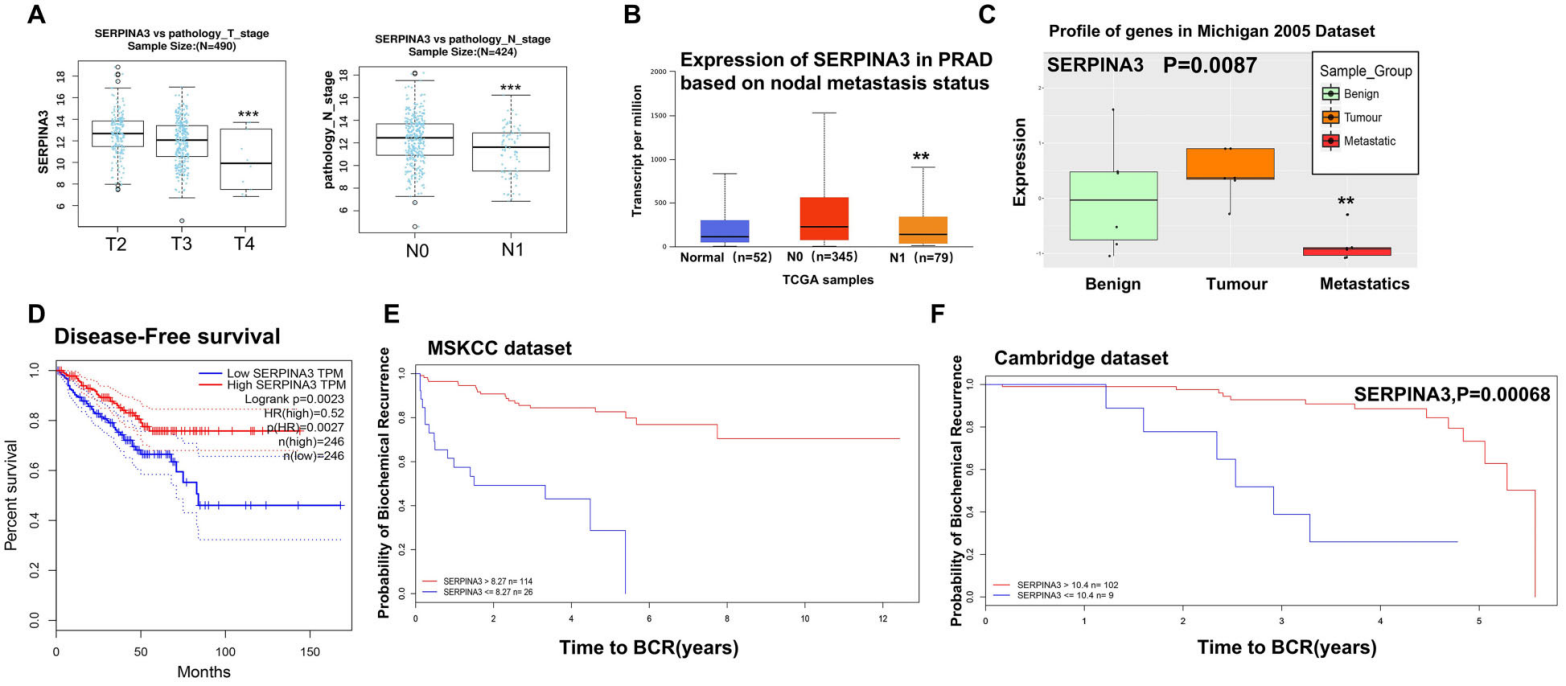
Impact of low SERPINA3 expression on the metastasis of prostate cancer and patient survival. **A**, LinkedOmics online platform was used to analyze the correlation between SERPINA3 and TNM staging in patients with prostate cancer from the TCGA-PRAD database. **B**, UALCAN online platform was used to analyze the correlation between SERPINA3 and TNM staging in patients with prostate cancer. **C**, Michigan 2005 prostate cancer dataset was analyzed to investigate the relationship between SERPINA3 expression and tumor metastasis. **D**, GEPIA online platform was used to analyze the relationship between the expression of SERPINA3 and disease-free survival in patients with prostate cancer from the TCGA-PRAD database. **E** and **F**, MSKCC and Cambridge datasets were analyzed to examine the association between SERPINA3 expression and the probability of freedom from biochemical recurrence (BCR) in patients with prostate cancer. Data are reported as median and interquartile range. **P<0.01; ***P<0.001; Mann-Whitney U test. Survival curves were estimated using the Kaplan-Meier method.

To elucidate whether the expression of SERPINA3 is associated with PCa metastasis and patient survival, we first analyzed the clinical significance of SERPINA3 expression in the LinkedOmics database, which showed that low expression of SERPINA3 was significantly negatively correlated with clinical progression and lymph node metastasis in PCa (Figure 3A). In the UALCAN database, a significant negative correlation was also found between the expression of SERPINA3 and clinical PCA TNM analysis (Figure 3B). Furthermore, the Michigan 2012 PCa dataset confirmed that the expression of SERPINA3 in tissues of patients with metastatic PCa was significantly lower than that of normal patients and those with non-metastatic PCa (Figure 3C). When examining the association between SERPINA3 expression and the survival of patients with PCa, we found in the Gene Expression Profiling Interactive Analysis database that low expression of SERPINA3 may significantly reduce patient survival rates. Similarly, the MSKCC and Michigan PCa datasets showed that low expression of SERPINA3 significantly reduced the overall survival of patients with PCa (Figure 3D-F). Based on the aforementioned bioinformatics results, it may be preliminarily concluded that low expression of SERPINA3 could be related to PCa progression and reduced patient survival time.

### Overexpression of SERPINA3 significantly reduced the malignant progression of PCa *in vitro* and *in vivo*

**Figure 4:**
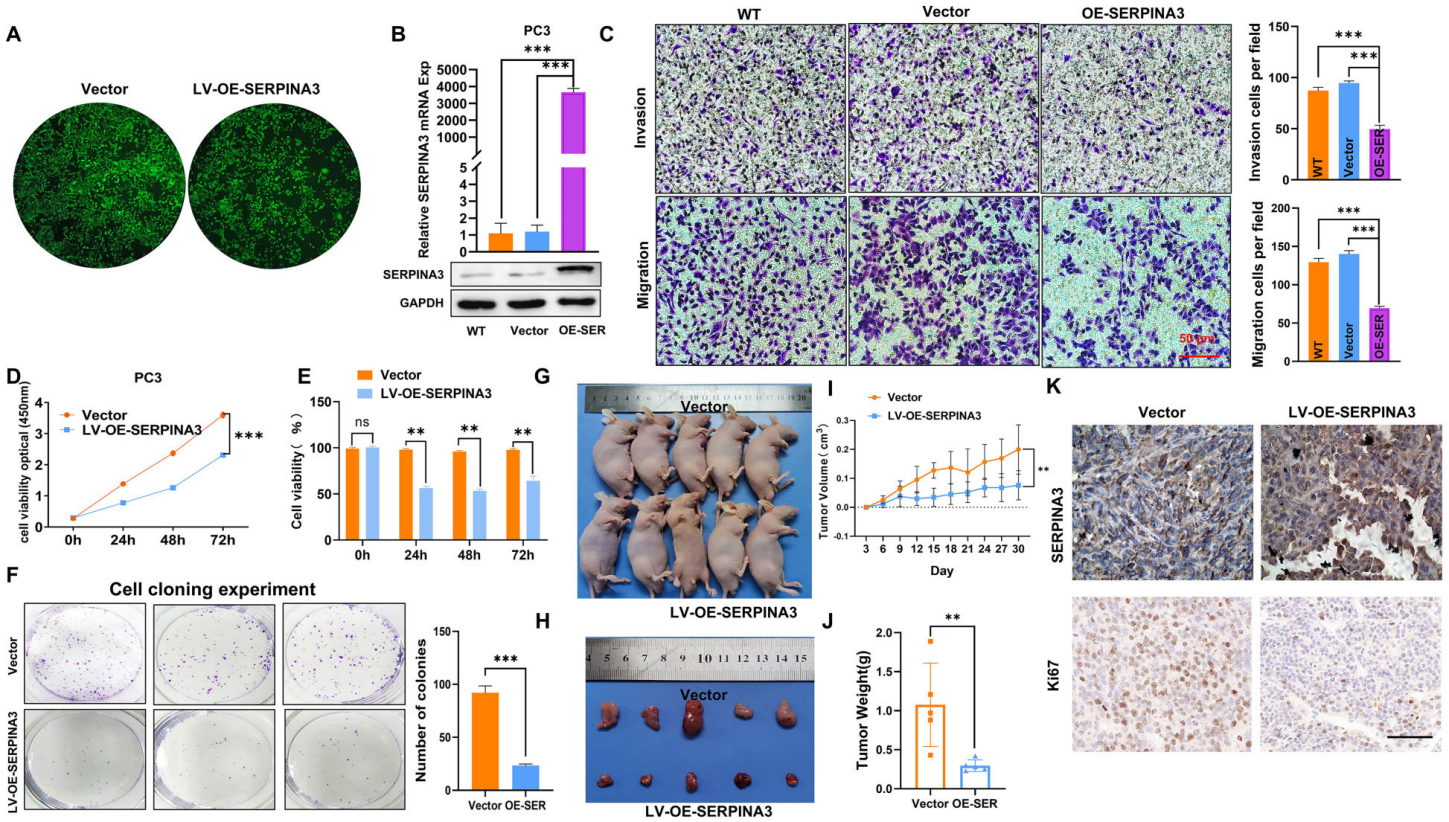
Overexpression of SERPINA3 inhibits the malignant progression of prostate cancer. **A**, GFP-fluorescence tagged SERPINA3-overexpressing lentivirus was stably infected into the PC3 cell line. **B**, Western blotting and RT-qPCR were conducted to detect the overexpression of SERPINA3. **C**, Transwell assay was performed to verify the change in invasive and migratory capabilities of PC3 cells following the overexpression of SERPINA3. WT: wild type. **D**, The CCK-8 assay confirmed that the overexpression of SERPINA3 in PC3 cells altered their proliferative capacity, as demonstrated by absorbance measurements at 0, 24, 48, and 72 h. **E**, Cell viability rate. **F**, Colony formation assay was conducted to assess the colony-forming ability of cells following overexpression of SERPINA3. **G**, Subcutaneous tumor experiment in nude mice was performed to investigate the effect of SERPINA3 overexpression on tumor proliferation (n=5 in each group). **H**, Subcutaneous tumors isolated from mice. **I**, Growth volume of subcutaneous tumors in Vector group and OE-SERPIAN3 group mice for 30 days. **J**, Weight of subcutaneous tumors in mice. **K**, Representative images of immunohistochemical staining were used to assess the expression of SERPINA3 and Ki67. Data are reported as means±SE and were analyzed by two-tailed unpaired Student's *t*-test. **P<0.01, ***P<0.001. ns: not significant. Scale bar, 50 μm.

To validate the function of SERPINA3, PC3 cells were selected for lentivirus infection to induce overexpression of SERPINA3, and the overexpression efficiency was verified at RNA and protein levels (Figure 4A and B). Subsequently, a transwell assay was conducted on PC3 cells stably overexpressing SERPINA3, which confirmed that overexpression of SERPINA3 could inhibit the invasion and migration of PC3 cells (Figure 4C). In terms of cell proliferation, the CCK8 assay also provided evidence that overexpression of SERPINA3 inhibited cell proliferation (Figure 4D and E). Additionally, the colony formation assay demonstrated that overexpression of SERPINA3 in PC3 cells could inhibit the formation of cell colonies (Figure 4F). The *in vivo* experiments reinforced the conclusions drawn from the cell experiments, demonstrating that overexpression of SERPINA3 could inhibit tumor proliferation (Figure 4G and H). Following tumor transplantation, mice overexpressing SERPINA3 exhibited significantly reduced tumor volume growth and final tumor weight (Figure 4I and J). In addition, two groups of subcutaneous tumor tissues were collected for immunohistochemical observation of Ki67 staining to assess proliferation. The results indicated that the tumor proliferation ability of mice in the SERPINA3-overexpressing group was significantly weakened (Figure 4K). These experimental results indicated that overexpression of SERPINA3 may significantly inhibit the proliferation, invasion, and migration of PCa.

### SERPINA3 mediated activation of the IL-17/TNF**α** signaling pathway by regulating the expression of CXCL2

**Figure 5:**
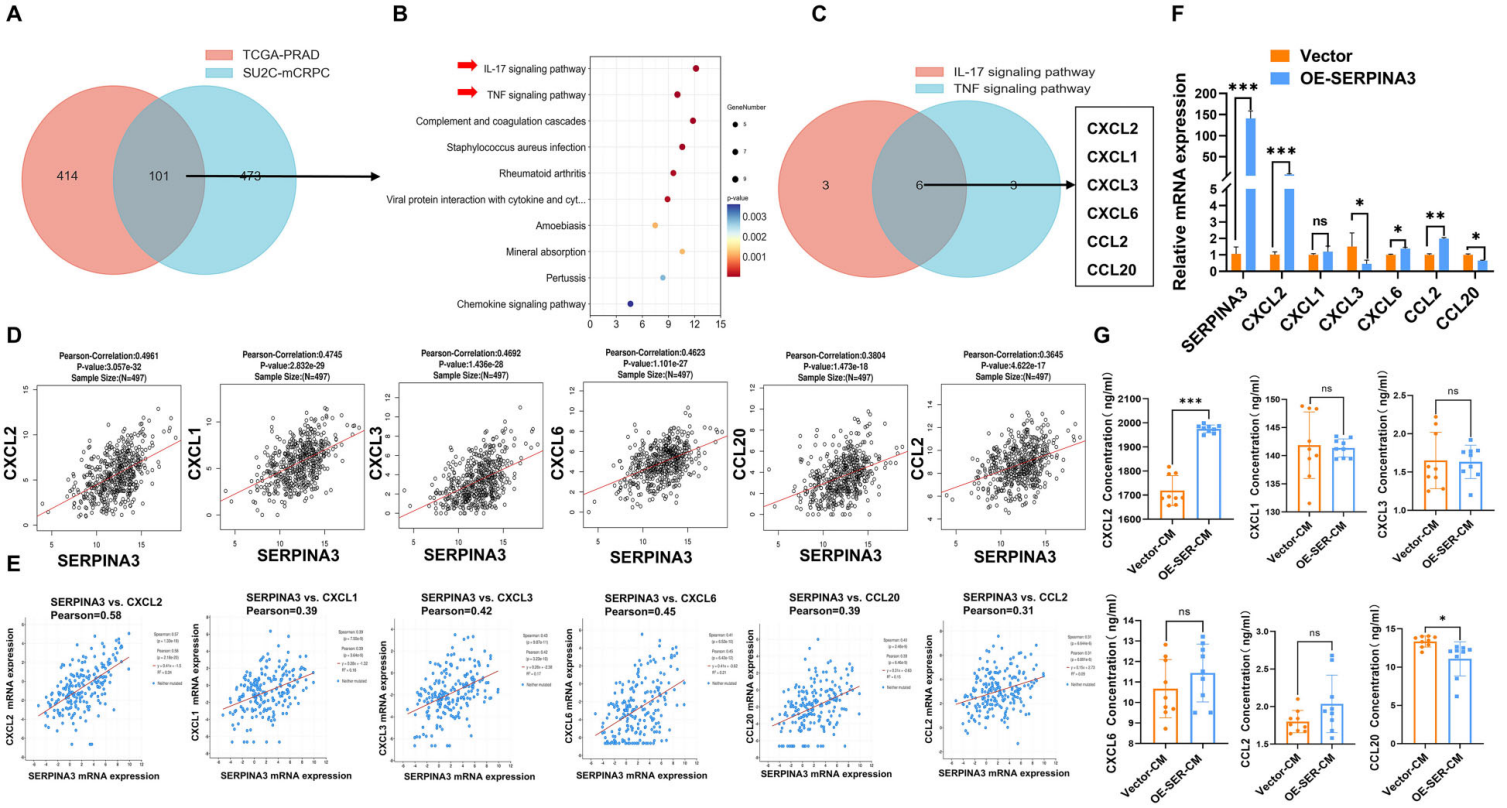
SERPINA3 mediates activation of the interleukin (IL)-17/tumor necrosis factor (TNF)α signaling pathway by regulating the expression of CXCL2. **A**, Venn intersection analysis was performed on the differentially expressed genes associated with SERPINA3 in TCGA-PRAD and SU2C-mCRPC datasets. **B**, KEGG enrichment analysis of signaling pathways associated with the 101 intersecting genes identified in **A**. **C**, Venn intersection of genes enriched in both the IL-17 signaling pathway and the TNFα signaling pathway. **D**, TCGA-PRAD database analysis was conducted to assess the correlation between SERPINA3 and CXCL1, CXCL2, CXCL3, CXCL6, CCL2, and CCL20. **E**, SU2C-mCRPC cohort was used for assessing the correlation between SERPINA3 and CXCL1, CXCL2, CXCL3, CXCL6, CCL2, and CCL20. **F**, RT-qPCR experiments were performed to detect the expression of CXCL1, CXCL2, CXCL3, CXCL6, CCL2, and CCL20 in PC3 cells overexpressing SERPINA3. **G**, ELISA experiments were performed to detect the concentrations of CXCL1, CXCL2, CXCL3, CXCL6, CCL2, and CCL20 in the supernatants of PC3 cells from both the Vector group and the SERPINA3-overexpressing group. Data are reported as means±SE and were analyzed by two-tailed unpaired Student's *t*-test. *P<0.05, **P<0.01, ***P<0.001. ns: not significant.

To validate the downstream signaling pathways affected by SERPINA3, a Venn diagram intersection analysis of DEGs related to SERPINA3 was performed in the SU2C-mCRPC and TCGA-prostate adenocarcinoma (PRAD) databases. There were 101 DEGs that were commonly enriched in both datasets (Figure 5A). KEGG pathway enrichment analysis was then performed on the intersecting genes and the two signaling pathways with the lowest P-values were the IL-17 signaling pathway and the TNFα signaling pathway (Figure 5B). Furthermore, a Venn diagram analysis was performed on the DEGs in the IL-17 and TNFα signaling pathways, and six genes were found to be enriched in both pathways (Figure 5C). The correlations between SERPINA3 and these six DEGs were then analyzed in both TCGA-PRAD and SU2C-mCRPC databases, and the correlation between SERPINA3 and CXCL2 was the strongest (Figure 5D and E). Furthermore, the expression of these six cytokines was detected in PC3 cells overexpressing SERPINA3, and the results revealed that the upregulation of CXCL2 expression was the most significant (Figure 5F). ELISA experiments were then performed using the supernatants from PC3 cells in the Vector and SERPINA3-overexpressing groups to detect the concentrations of the six cytokines. The results showed that CXCL2 secretion was significantly more increased from the supernatants of SERPINA3-overexpressing cells compared with those of Vector cells (Figure 5G). These experimental results suggested that SERPINA3 may exert its biological effects on tumors by regulating CXCL2, thereby influencing the IL-17 and TNFα signaling pathways and affecting the biological phenotype of tumors.

### SERPINA3 inhibited PCa progression by promoting the infiltration of M1 macrophages

**Figure 6:**
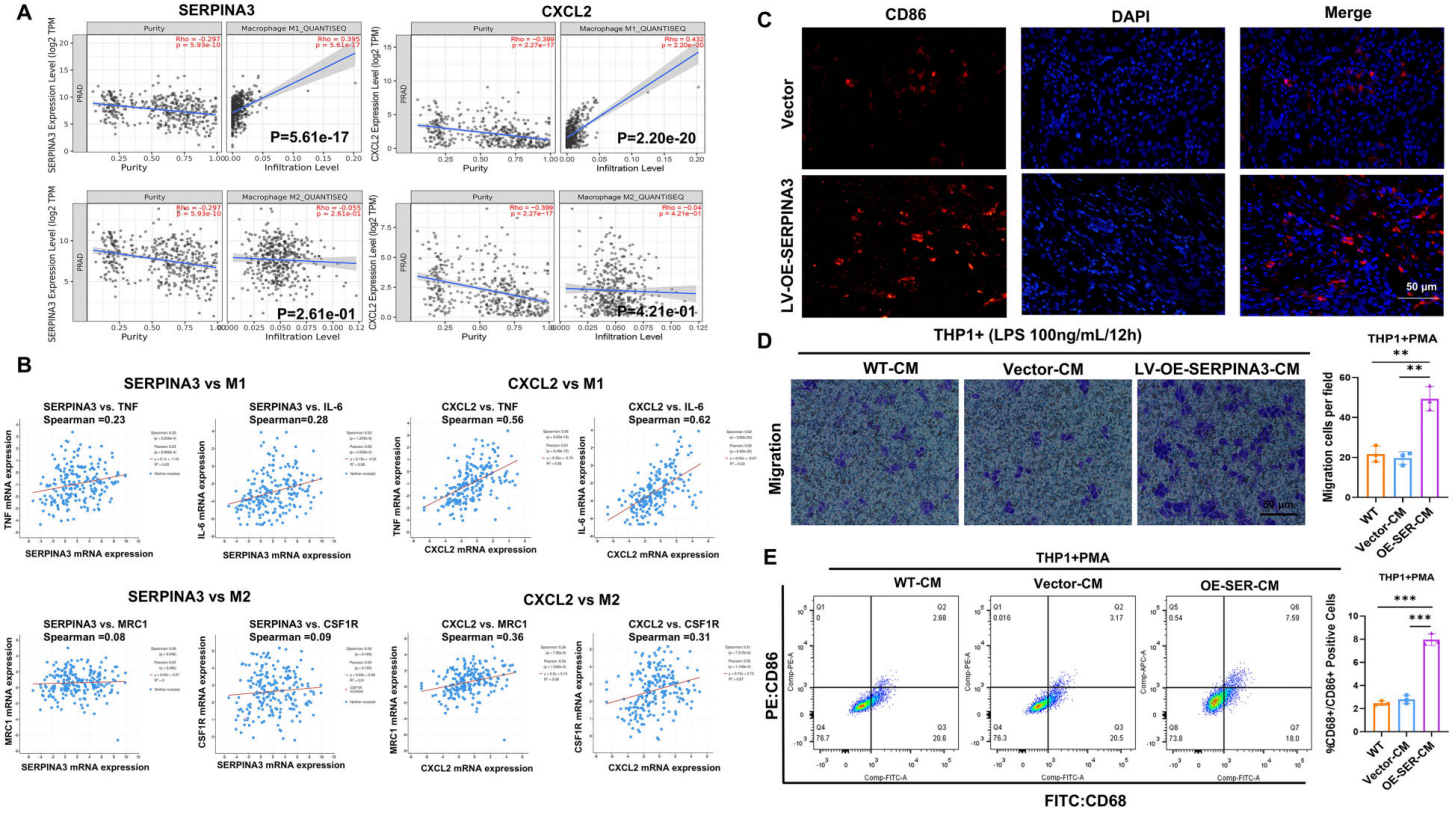
SERPINA3 inhibited the progression of prostate cancer by promoting the infiltration of M1 macrophages. **A**, Timer2.0 database was used to analyze the correlation of SERPINA3 and CXCL2 expression with the infiltration of M1/M2 macrophages in prostate cancer using the QUANTISEQ computational method. **B**, SU2C-mCRPC cohort was separately analyzed to assess the correlation of SERPINA3 and CXCL2 with the M1 macrophage markers (tumor necrosis factor (TNF), interleukin (IL)-6) and the M2 macrophage markers (MRC1, CSF1R). **C**, Representative immunofluorescence detection of mouse tumors from Figure 4H showing the staining of the M1 macrophage marker CD86. Scale bar, 50 μm. **D**, Transwell assay was conducted to assess the recruitment ability of M1 macrophages by the conditioned medium from PC3 cells in the wild-type group, Vector group, and the group overexpressing SERPINA3. The quantitative results are shown on the right. **E**, Flow cytometry was conducted to assess the impact of conditioned medium from PC3 cells in the wild-type group, Vector group, and the group overexpressing SERPINA3 on the polarization of M1 macrophages (proportion of CD68^+^ and CD86^+^ cells). The quantitative results are shown on the right. QUANTISEQ is based on the deconvolution algorithm and utilizes the RNA-seq data from bulk samples to predict the composition of different types of immune cells in tumor samples. It supports 10 types of immune cells, including B cells, classically activated macrophages (M1), alternately activated macrophages (M2), monocytes, neutrophils, NK cells, non-regulatory CD4^+^ T cells, CD8^+^ T cells, dendritic cells and other uncharacterized cells. All data are reported as means±SE. **D** and **E,** One-way ANOVA was used for statistical analysis. **P<0.01, ***P<0.001. CM: conditioned medium; NK: natural killer; WT: wild type.

It is known that both the IL-17 and TNFα signaling pathways are associated with M1 macrophage polarization, and M1 macrophages are known to be a key factor in exerting tumor-suppressive effects in the tumor microenvironment (10-13). The present study hypothesized that SERPINA3 may activate the IL-17 and TNFα signaling pathways by influencing the expression of CXCL2, thereby affecting the recruitment of M1 macrophages and inhibiting PCa progression. Therefore, the correlation of SERPINA3 and CXCL2 expression with M1/M2 polarized macrophage infiltration in PCa was validated using QUANTISEQ methods in the Timer2.0 tumor immune infiltration-related database. The results showed that the correlation of SERPINA3 and CXCL2 expression with M1 macrophage infiltration was significantly greater than that with M2 macrophage infiltration (Figure 6A). Subsequently, the correlation of SERPINA3 and CXCL2 expression with M1/M2 macrophage marker expression was analyzed in the mCRPC database, which showed that the correlation between SERPINA3 and M1 macrophage markers (TNF and IL-6) was significantly higher than that between SERPINA3 and M2 macrophage markers (MRC1 and CSF1R) (Figure 6B).

Considering the aforementioned results, the expression of SERPINA3 in PCa may be associated with the infiltration of M1 macrophages. Therefore, immunofluorescence staining was performed on the subcutaneous tumors depicted in Figure 4. The results showed a significant increase in the infiltration of M1 macrophages (CD86 is a classical marker of M1 macrophages) in the group overexpressing SERPINA3 (Figure 6C). Additionally, *in vitro* experiments were designed to validate the relationship between SERPINA3 expression in tumor cells, and the recruitment and polarization of macrophages. THP-1 cells were induced to differentiate into M0 macrophages using PMA, followed by LPS (100 ng/mL) treatment for 12 h to induce M1 polarization. Subsequently, a transwell assay was performed, which showed that the conditioned medium from PC3 cells overexpressing SERPINA3 enhanced the recruitment of M1 macrophages (Figure 6D). Subsequently, THP-1 cells were induced into M0 macrophages, and then co-cultured with conditioned medium from PC3 cells overexpressing SERPINA3 or from WT and Vector control cells. Flow cytometry was used to detect M1 macrophage polarization markers (CD68^+^, CD86^+^). It was found that macrophages in the SERPINA3-overexpressing group were significantly polarized towards M1 (Figure 6E). These experimental results indicated that SERPINA3 inhibited the progression of PCa by promoting the recruitment and M1 polarization of macrophages.

## Discussion

In our study, we reported that SERPINA3 played a crucial role in the progression of CRPC. While previous cancer research has focused on SERPINA3 as an oncogenic factor, our research revealed the heterogeneity of SERPINA3 in cancer. Through bioinformatics analysis and both *in vitro* experiments, we confirmed that SERPINA3 can significantly inhibit the progression of prostate cancer. Mechanistically, we found that SERPINA3 may influence the expression of CXCL2 to activate the IL-17 and TNFα signaling pathways, thereby mediating the recruitment of M1 macrophages in the tumor microenvironment and exerting an inhibitory effect on prostate cancer progression.

CRPC is a lethal stage of PCa, and the specific mechanisms underlying its progression remain unclear, despite relevant research. However, the latest viewpoint proposes that the recruitment of macrophages in the tumor microenvironment is a crucial driving force promoting the progression of PCa in patients with CRPC. Yet, the exact reasons for the recruitment of macrophages in the tumor microenvironment still require further investigation. In the present study, low expression of SERPINA3 was identified to be potentially associated with CRPC through analysis of CRPC datasets. However, current research on SERPINA3 has indicated that it is upregulated in various types of cancer, such as glioblastoma, colorectal cancer, endometrial cancer, breast cancer, and melanoma, and can participate in tumor progression by regulating signaling pathways, such as PI3K/AKT (7-9,14-18). Nonetheless, the function of SERPINA3 remains partially understood, and its role in CRPC has yet to be explored. In the present study on SERPINA3, it was discovered that its antitumor effect was primarily achieved through the recruitment of numerous M1 macrophages into the tumor microenvironment, which represents a novel viewpoint. Additionally, it was suggested that CXCL2 may act as a downstream mediator of SERPINA3, facilitating the recruitment of macrophages and promoting their polarization towards the M1 phenotype.

CXCL2, also known as macrophage inflammatory protein-2 or growth-regulated protein-β, belongs to the CXC family of chemokines (19). Current research has suggested that CXCL2 is associated with the progression of various tumors, such as liver cancer, breast cancer, colon cancer, and lung cancer (20-23). Moreover, there is growing interest in the role of CXCL2 in mediating the polarization of macrophages towards the M1 phenotype in the tumor microenvironment (24). However, there is no study confirming the promotion of tumor progression in PCa through CXCL2-mediated polarization of macrophages towards the M1 phenotype. Due to the strong heterogeneity of prostate cancer, many studies currently suggest that during the progression of prostate cancer, the proportion of macrophages in the tumor microenvironment significantly increases and may be an important factor in promoting the progression of prostate cancer (25). Furthermore, the different polarization states of macrophages have varying effects on tumors: M2-polarized macrophages can promote tumor progression, while M1-polarized macrophages inhibit it (12). Interestingly, in Figure 5A-C, we unexpectedly found through KEGG pathway enrichment and Venn intersection analysis of SERPINA3-related differentially expressed genes in both the TCGA-prostate cancer database and the SU2C-metastatic prostate cancer database that the IL-17 and TNF signaling pathways were the top two enriched pathways. Current research has confirmed that these two signaling pathways can promote the M1 polarization of macrophages. Therefore, we will further analyze the infiltration levels of M1 macrophages in tumor tissues to determine whether SERPINA3 inhibits the progression of prostate cancer by promoting the increased infiltration of M1 macrophages into the tumor microenvironment. However, there are still some limitations, since the specific mechanisms by which SERPINA3 regulates CXCL2 and its related pathways remain unconfirmed.

In summary, based on previous research, the progression of PCa is closely associated with a substantial infiltration of macrophages. SERPINA3 serves a crucial role in inhibiting the progression of PCa. To the best of our knowledge, the present study is the first to propose that SERPINA3 can mediate the M1 polarization of macrophages by regulating the expression of CXCL2, thus suppressing the progression of PCa. This discovery provides new theoretical support for the tumor microenvironment-mediated progression of PCa and may create new opportunities for subsequent clinical treatments. Interestingly, as SERPINA3 functions as a plasma protease, it may also serve as a potential biomarker for blood-based detection of PCa, enabling precise monitoring of tumor progression and postoperative recurrence in patients with PCa. These findings hold potential clinical significance.
